# Exploration of the Optimal Treatment Modality for Vitreoretinal Lymphoma: A PRISMA Compliant Meta‐Analysis and Systematic Review

**DOI:** 10.1002/cam4.71092

**Published:** 2025-07-30

**Authors:** Jing Gao, Li‐wei Lv, Jing Yang, He‐nan Wang, Liang Wang

**Affiliations:** ^1^ Department of Hematology Beijing Tongren Hospital, Capital Medical University Beijing China

**Keywords:** systematic review and meta‐analysis, treatment strategies, vitreoretinal lymphoma

## Abstract

**Background:**

Vitreoretinal lymphoma (VRL) is regarded as a subtype of primary central nervous system lymphoma (PCNSL). Hence, extending progression‐free survival (PFS) is crucial for enhancing the prognosis of VRL patients. Nevertheless, a lack of standard treatment options currently exists for VRL. This systematic review aims to explore the most optimal treatment strategy.

**Methods:**

The methods for this systematic review and meta‐analysis adhered to PRISMA guidelines and followed a protocol registered on the PROSPERO registry. A search was conducted on PubMed, Embase, and Scopus up to October 14, 2023, using predefined search terms. Primary endpoints included overall response rate (ORR) and complete response rate (CRR), while secondary endpoints comprised overall survival (OS) and progression‐free survival (PFS).

**Results:**

Thirty‐seven studies comprising 801 patients were included in the meta‐analysis. The pooled CRR was 85%, and the ORR was 93%. The pooled 1‐year PFS was 83%, and the 2‐year PFS was 58%. The 1‐year OS was 92%, and the 2‐year OS was 80%. The combined median PFS was 22.87 months, and the median OS was 51.19 months. Survival analysis of the extracted data showed significant associations between PFS and OS with systemic therapy (*p* = 0.00098 and *p* = 0.0091) and multi‐strategy combination therapy (*p* = 0.0081 and *p* = 0.007); however, age, gender, and bilateral involvement exhibited no significant relationship (*p* > 0.05).

**Conclusions:**

In conclusion, while existing treatment strategies have led to higher remission rates and longer OS for VRL patients, PFS remains suboptimal. The primary focus of future clinical and basic research will be to explore effective treatment strategies for controlling disease recurrence or progression.

**Trial Registration:** This meta‐analysis was registered in the international prospective register of systematic reviews (PROSPERO) (CRD42023400305).

## 1 Introduction

Vitreoretinal lymphoma (VRL) is a subtype of central nervous system lymphoma (CNSL), predominantly of B‐cell lymphoma nature. VRL is classified as a subtype of non‐Hodgkin lymphoma that predominantly affects the vitreous body or retina [1]. According to whether malignant lymphocytes first appear in the vitreous or retina, they are divided into primary and secondary types [1]. Primary vitreoretinal lymphoma (PVRL) refers to lesions that first occur in the vitreous or retina and do not involve the central nervous system or other systems [2]. Secondary vitreoretinal lymphoma (SVRL) refers to secondary to CNSL or systemic lymphoma [3]. The long‐term prognosis of VRL is often unsatisfactory, with approximately 65%–85% of patients eventually dying from CNSL [4].

As a rare disease, VRL lacks large‐scale epidemiological data, making it difficult to accurately estimate its incidence rate. Current available information is based on localized reports. Levasseur et al. found that the incidence rate of VRL in British Columbia in 1990–2010 was approximately 0.047 per 100,000 people per year, primarily affecting elderly female patients [5]. A recent epidemiologic study found that the overall incidence of VRL in the United States was 0.23 per 1 million, showing a steady upward trend from 1992 to 2018, and typically occurring in those aged 60 years and older [6].

VRL is challenging to diagnose because it often masquerades as uveitis. Diagnosis currently relies on vitrectomy, vitreous cytokine testing, imaging (such as angiography or OCT), and testing for the MYD88 mutation, all contributing to the diagnosis of VRL [2]. To date, there is no standardized and uniform international treatment for VRL. Treatment modalities vary widely, including methotrexate‐based local or systemic therapy, local or whole‐brain radiotherapy, and combined chemotherapy with hematopoietic stem cell transplantation (IC + ASCT). Additionally, drugs such as BTK inhibitors, temozolomide, and rituximab have begun to be considered as potential therapeutic agents in ongoing exploration; however, their efficacy and safety in VRL treatment remain undetermined.

In conclusion, the treatment of VRL remains a challenging issue, necessitating further research and clinical practice to explore more effective therapeutic strategies. This paper aims to conduct a systematic review to discuss the optimal treatment options for VRL, providing scientific evidence for clinical practice and fostering further advancements and innovations in the field.

## 2 Method

This systematic review and meta‐analysis adhered to a previously published protocol registered on the PROSPERO registry (CRD42023400305). Following the PRISMA (Preferred Reporting Items for Systematic Reviews and Meta‐Analyses) guidelines, an exhaustive literature search was executed to identify articles published up to October 14, 2023, on PubMed, Embase, and Scopus [7].

### 2.1 Search Strategy and Inclusion Exclusion Criteria

The search algorithm involved combining terms related to intraocular lymphoma, treatments, and clinical trials, as depicted in Table S1. Limits were applied to exclude reviews, case reports, and animal experiments. Inclusion criteria for this review included: (1) clinical studies; (2) studies published in English; (3) patients diagnosed with VRL or IOL; (4) prospective and retrospective studies on treatment; (5) studies reporting extractable endpoints, such as overall response rate (ORR), complete response (CR), partial response (PR), and survival data; (6) relevant conference abstracts. Simultaneously, studies meeting any of the following conditions were excluded from this review: (1) duplicate literature; (2) reviews, case reports, comments, cellular or animal studies; (3) non‐therapeutic or diagnostic studies; (4) manuscripts lacking original data; (5) updates on previous results; (6) studies involving patients with lymphoma localized outside the ocular region. In addition, for studies involving mixed populations, this paper will also extract and analyze any available VRL data included, such as relevant treatment effects and survival rates.

### 2.2 Study Endpoints and Screening Process

Complete remission (CR) is defined as no evidence of residual disease in vitreous or retina; partial remission (PR) is defined as a reduction of more than 50% in vitreous or retinal lesions compared to baseline, as confirmed by ophthalmologic examinations such as Optical Coherence Tomography; progressive disease (PD) is defined as a worsening of ocular manifestations or the development of new ocular pathology; and if none of the above is present, then it is stable disease (SD). The primary endpoints comprised the ORR and CRR. ORR denotes the proportion of patients achieving complete and partial responses, expressed as a percentage of the entire study cohort, while CRR indicates the proportion of patients achieving a complete response as a percentage of the total study population. Secondary endpoints encompassed OS and PFS. OS was defined as the duration from a specified reference point (e.g., initiation of treatment) to death from any cause, whereas PFS was defined as the duration from a specified time point (e.g., initiation of treatment) to disease progression or death from any cause.

The study selection process can be broadly divided into two stages. Initially, within the available literature, two researchers, Jing Gao and Li‐wei Lv, independently screened the title and abstract of each article for suitability for inclusion in the meta‐analysis, removing duplicates. Subsequently, the full text of studies meeting the criteria established in the first stage was compared by the two researchers, resolving any discrepancies through discussion or consultation with a third researcher (Lang Wang).

### 2.3 Data Extraction and Analysis

Two authors, Jing Gao and Li‐wei Lv, independently conducted data collection for eligible studies using an Excel sheet (refer to Table 1), which included the first author's name, year of publication, country, study design (type and phase), study period, median follow‐up time, disease state, sample size, median age, patient gender, primary intervention, survival data, adverse events (AEs), and relapses (central and ocular). Disagreements arising from data collection were resolved through joint discussion with the third author, Lang Wang. The OR, CR, OS, and PFS data extracted from the literature were initially combined using forest plots, incorporating median survival time, 1‐year survival time, and 2‐year survival time. Additionally, survival data were obtained from Kaplan–Meier curves using Engauge Digitizer software version 10.8, followed by analyses using the Kaplan–Meier method and the log‐rank test to plot survival curves for comparison. Heterogeneity across studies was assessed using Cochran's Q test and the *I* ^2^ statistic. Fixed‐effects models were employed when heterogeneity was not significant (*I* ^2^ < 50% or *p*‐value > 0.1), while random‐effects models were utilized when heterogeneity was significant. Publication bias was examined using the Egger regression test for funnel plot asymmetry; a *p*‐value of less than 0.05 indicated the presence of statistically significant publication bias. All statistical analyses were conducted using R version 4.1.1 (R Foundation for Statistical Computing, Vienna, Austria).

**TABLE 1:** Baseline clinical characteristics of included retrospective studies.

Study	Year	Country	Design	Study period	Median follow‐up time, months (range)	Disease status	Sample size	Median age, years (range)	Gender male/female	Primary intervention	Survival data	AEs	Relapses (central, ocular)
Ferreri, et al.	2002	Italy	Retrospective	1980–1999	/	Intraocular lymphoma (IOL)	22	54 (16–74)	15/7	HD MTX/intrathecal chemotherapy			15/21, 9/21
Isobe, et al.	2006	Japan	Retrospective	1990/1–2005/2	/	Primary intraocular lymphoma (PIOL)	15	68 (38–84)	6/9	radiation therapy (RT)	1‐year OS: 87% (70%–100%) 2‐year OS: 74% (47%–100%) 1‐year DFS: 67% (43%–91%) 2‐year DFS: 58% (33%–84%)		
Jahnke, et al.	2006	Germany	Retrospective multicentre	1999–2004	10.25 (1–49)	IOL	22	64 (38–83)	11/11	Ifosfamide (IFO) or trofosfamide (TRO) based chemotherapy	mPFS: 10 mOS: 22.5		
Grimm, et al.	2007	/	Retrospective	1977–2005	/	HIV negative, immunocompetent PIOL	83	63 (24–85)	36/47	combined therapy			29/47, 21/47
Sorensen, et al.	2013	United States	Retrospective	/	/	Primary vitreoretinal lymphoma (PVRL)	12	/	/	Bilateral RT + MTX‐based systemic intensive chemotherapy			
Zierhut, et al.	2014	German	Retrospective	/	/	PIOL	5	55.2 (40–74)	2/3	Peripheral blood stem cell transplantation (PBSCT)			
Teckie, et al.	2014	United States	Retrospective	1999–2011	25 (2–150)	PIOL	18	64 (32–82)	7/11	RT			7/18, 4/18
Riemens, et al.	2015	Europe	Retrospective	1991/1–2012/12	49 (15–246)	PVRL	78	58 (38–86)	34/44	ocular and/or extensive systemic treatment			28/78, 17/78
Lee, et al.	2015	Korea	Retrospective multicentre	2007/12–2014/6	/	IOL	20	59 (34–76)	13/7	Intraocular MTX and/or MTX‐based systemic intensive chemotherapy	mPFS: 19.7 (8.7–30.7) 3OS: 0.751		
Ma, et al.	2016	China	Retrospective	2003/1–2013/12	40.2 (4.4–123.3)	PIOL	19	57 (39–77)	6/13	Intravitreous MTX + systemic high‐dose MTX	mPFS:19.7 (13.8–25.5) 5‐year OS: 0.558 mOS: 72.4	Superficial punctate keratitis [8] cataracts [7] Glaucoma [3] Sterile endophthalmitis [2] Hepatotoxicity [9] Nephrotoxicity [3]	6/19, 9/19
Cheah, et al.	2016	United States	Retrospective database	2007/10–2015/4	50.4 (21.6–91.2)	PIOL	11	66 (48–72)	2/9	R‐MPV + RT + HDAraC	mPFS:45.6	Grade 3 cataracts [6] Grade 3 neutropenia [3] Grade 3 keratitis [3]	
Kim, et al.	2016	United States	Retrospective	1994–2010	29 (10.2–96.4)	Primary and concurrent IOL	22	65	8/14	MTX‐based systemic intensive chemotherapy + whole‐brain radiotherapy	mPFS: 34 mOS: 43.4		7/22, 5/22
Klimova, et al.	2018	Czech Republic	Retrospective	2004–2016	56 (3–166)	PVRL and Primary CNS lymphoma (PCNSL)	10	61 (48–77)	/	Intravitreous MTX + systemic high‐dose MTX + intrathecal MTX	5OS:0.89 5PFS:0.45		4/10, 1/10
de la Fuente, et al.	2019	United States	Retrospective	2005/2–2018/8	68 (17–154)	PVRL	12	64 (38–81)	7/5	Bilateral RT + MTX‐based systemic intensive chemotherapy	5‐year PFS: 64% (95%, 29.8%–85.1%) 5‐year OS: 80% (40.9%–94.6%)		4/12
Castellino, et al.	2019	United States	Retrospective	1990–2018	33.6 (1.2–175.2)	primary, concurrent, secondary Vitreoretinal lymphoma (VRL)	69	65 (36–85)	34/35	Intraocular therapy and/or MTX‐based systemic intensive chemotherapy	ALL mFFS:21.6 (10.8–33.6) PVRL mFFS:31.2 (13.2–62.4)		19/51, 23/51
Lim, et al.	2019	United States	Retrospective	2015/9–2018/11	12	VRL	6	70	3/3	Intravitreal melphalan			
Baron, et al.	2020	France	Retrospective	/	42 (9–115)	PVRL	21	75 (35–90)	/	Temozolomide		Grade 3 anemia and vomiting [3] Grade 4 neutropenia and thrombocytopenia [1]	5/21
Anthony, et al.	2021	United States	Retrospective	/	26 (3–49)	PVRL	7	69 (56–85)	4/3	Intravitreous MTX			
Lam, et al.	2021	French	Retrospective	2011/1–2018/3	61 (50–71)	PVRL	59	70 (39–88)	14/45	MTX‐based systemic intensive chemotherapy	mPFS: 18 (12–37) mBFS: 73 (48‐NR) mOS: 75 (68‐NR) 5‐year OS: 0.67 (0.53–0.8)	Febrile neutropenia [3] Grade III‐IV hepatic cytolysis [4] Grade III‐IV renal toxicity [4]	22/59, 17/59
Wang, et al.	2021	China	Retrospective	2020/5‐?	7.5 (4–15)	VRL	11	61 (41–73)	4/7	Zanubrutinib Orelabrutin		Hypertension [1]	
Gozzi, et al.	2021	Italy	Retrospective	2006/1–2020/10	22 (9–58)	VRL	22	65 (55–72)	10/12	Intravitreous MTX and/or MTX‐based systemic intensive chemotherapy			
Gange, et al.	2022	United States	Retrospective	/	/	VRL	11	59 (42–83)	7/4	Intravitreal MTX or rituximab	MTX mOS:53.2 (41.8–64.6) Rituximab mOS:48.5 (24.7–72.4)		
Zhou, et al.	2022	China	Retrospective	2009/4–2019/8	30.55 (12–73)	VRL	40	62.5 (31–81)	14/26	Intravitreous MTX	mPFS: 20.82 (14.64–27.01) mOS: 29.29 (16.16–42.41)		7/40, 3/40
Hsu, et al.	2022	China	Retrospective	2013/1–2018/1	/	IOL	12	/	5/7	Intravitreous MTX + systemic high‐dose MTX	5‐year OS: 0.91	Keratitis [7] Grade 3 keratitis [6]	3/12, 3/12
Cheng, et al.	2023	China	Retrospective	2003–2018	103.5	VRL	32	63 (39–78)	10/22	Intravitreous MTX and systemic high‐dose MTX	5‐year OS: 0.733 10‐year OS: 0.589 mPFS: 20.3 (11.8–28.8) m CNS PFS: 69.5 (31.5–107.5)	Grade 3/4 neutropenia [4] Grade 3/4 thrombocytopenia [3] Grade 4 febrile neutropenia [2] Grade 3 hepatotoxicity [10]	13/32, 12/32
Mankuzhy, et al.	2023	United States	Retrospective	1990–2022	52	PIOL	29	/	/	RT	mOS: 64.8 (95% CI 46.8, 91.2) FFR: 8.4 (5–25)		
Min, et al.	2023	Korea	Retrospective	2013/10–2020/3	/	PIOL	21	65 (32–81)	10/11	Intravitreous MTX ± systemic high‐dose MTX	mPFS: 21.3 (9.5–36.7)		8/21, 12/21

## 3 Result

### 3.1 Study Selection and Characteristics

After conducting an initial search, we obtained 739 relevant records. Of these, 153 were excluded due to duplication. We meticulously scrutinized the titles and abstracts of the remaining 586 publications. Among these, 498 studies were excluded for not meeting the eligibility criteria: 40 were reviews, 105 were studies on various diseases, 180 were case reports, 70 were diagnostic studies, 11 were animal studies, and 92 were non‐therapeutic studies. We then conducted a full‐text examination of the remaining 88 records, resulting in the exclusion of 51 articles, primarily due to data extraction issues (43 articles) and updated results (8 articles). Ultimately, only 37 full‐text articles or conference abstracts met the evaluation criteria (Figure S1). Out of the 37 included studies, 10 were prospective and 27 were retrospective, all with a follow‐up duration ranging from 0.2 to 246 months, with a median follow‐up time of 29.5 months. The patients' ages ranged from 16 to 90 years, with a median age of 64 years. Gender information was available for 660 out of the total 801 patients, with 259 being male and 401 being female. The diverse range of interventions in these studies included intraocular drug injections (e.g., methotrexate, rituximab), high‐dose methotrexate systemic chemotherapy (HD‐MTX), targeted therapies like Bruton's tyrosine kinase (BTK) inhibitors, radiotherapy, and other modalities (e.g., intrathecal MTX injections, stem cell transplantation). Besides survival data, adverse events and central/ocular recurrences were documented, with 192 central recurrences observed among 515 patients (37.3%) and 142 ocular recurrences among 472 patients (30%). The characteristics of the included patients are presented in Table 1 and Table 2.

**TABLE 2:** Baseline clinical characteristics of included prospective studies.

Study	Year	Country	Design	Study period	Median follow‐up time, months (range)	Disease status	Sample size	Median age, years (range)	Gender male/female	Primary intervention	Survival data	AEs	Relapses (central, ocular)
Stefanovic, et al.	2010	United States	Prospective	/	44 (10–51)	PIOL	6	59.5 (55–71)	4/2	Bilateral RT + MTX‐based systemic intensive chemotherapy			
Taoka, et al.	2012	Japan	Prospective trial	2007/11–2009/12	32 (21–42)	PIOL	5	65 (43–72)	2/3	Intravitreous MTX + *R*‐MPV + rdWBRT		Grade 3/4 Neutropenia [4]	
Pleyer, et al.	2014	Germany	Prospective, non‐interventional multicenter study	2008/8–2013/8	20.2	PIOL	20	67	8/12	High‐dose Methotrexate (MTX)‐ or Ifosfamide‐based systemic chemotherapy			
Akiyama, et al.	2016	Japan	Single‐arm prospective study	2007/1–2013/12	29.5	PIOL	10	68.5	4/6	Intravitreous MTX + systemic high‐dose MTX	2‐year CLFS: 58.3% (23.0%–82.1%)	Grade 3 anemia [2] Grade 3 anorexia [1]	4/10, 2/10
Kaburaki, et al.	2017	Japan	One‐arm Prospective Trial	2008/8–2015/3	48.9 (15.3–95.1)	PIOL with or without CNS involvement	17	63 (43–72)	9/8	Intravitreous MTX + *R*‐MPV + rdWBRT	4‐year PFS: 72.7% (59.3%–86.1%) 4‐year OS: 88.9% (78.4%–99.4%)		1/11, 2/11
Soussain, et al.	2019	France	Prospective Multi‐center, Open‐label, Phase II trial	2015/9–2016/7	25.7 (0.7–30.5)	R/R PCNSL and PVRL	14	70 (52–81)	/	Ibrutinib	mPFS:22.7 (5.5‐NA) 1PFS:0.714 (0.513–0.995) 2PFS:0.49 (0.284–0.845)		
Hoang‐Xuan, et al.	2020	France	Prospective Multi‐center, Open‐label, Phase II trial	2017/7–2019/10	6.7 (0.2–27.4)	PCNSL and PVRL	9	72 (43–83)	/	Pembrolizumab monotherapy	mPFS:2.6 6‐month PFS: 29.8% 6‐month OS: 60.4%		
Zhang, et al.	2021	China	Prospective Single‐center, Open‐label, Phase II trial	2018/8–2020/1	18.3 (10.6–27.8)	PVRL	11	58 (48–70)	3/8	R2 + IV‐MTX Lenalidomide maintain		Grade 3 cataracts [6] Grade 3 neutropenia [1]	5/11, 3/11
Zhang, et al.	2022	China	Prospective Multi‐center, Open‐label, Phase II trial	2020/8–2022/1	12.4 (0.3–18.1)	PVRL	10	55 (39–70)	3/7	ZR (zanubrutinib + rituximab)	1‐year PFS: 68.5% (21.3%–92.2%)	Grade 1 bruising [4] Grade 2 hypermenorrhea [1] Grade 3 vitreous hemorrhage [1]	2/10
Guan, et al.	2022	China	Prospective Single‐center, Open‐label, Phase II trial	2020/10–2022/4	8.3 (2.5–21.4)	VRL	10	/	/	Ibrutinib Zanubrutinib Orelabrutinib		Grade 1 ecchymosis [3] Grade 2 arthralgia [1]	3/10, 2/10

### 3.2 Efficacy

#### 3.2.1 Remission Rates

**FIGURE 1:**
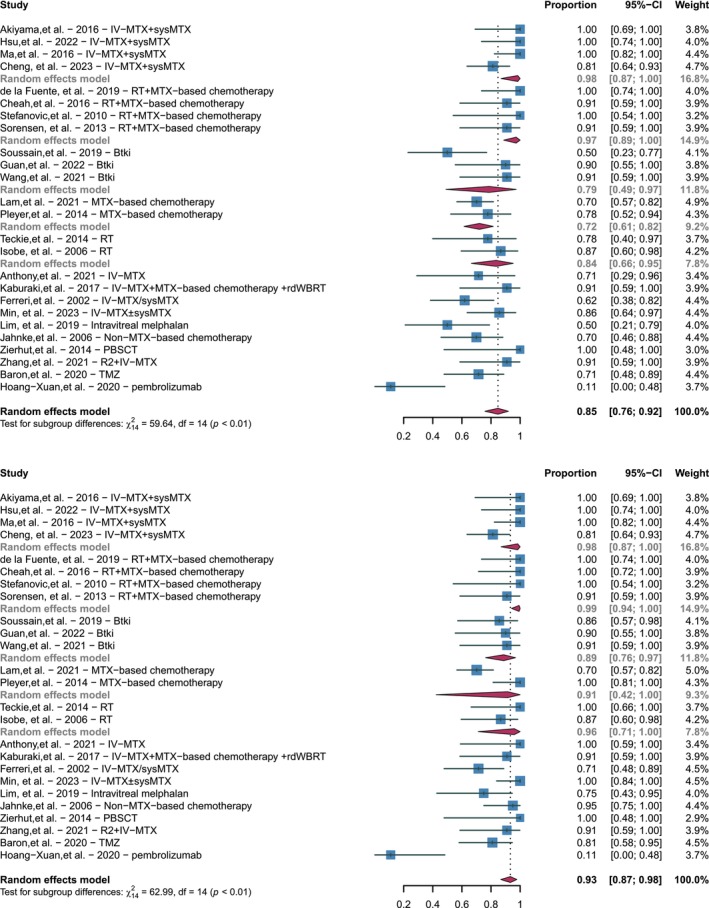
Forest plot showing remission rates of various treatment modalities for vitreoretinal lymphoma (VRL). (Top section) The pooled CRR of various studies. (Bottom section) The pooled ORR of various studies.

Remission rates for treating VRL were documented in 25 studies, encompassing CR, PR, and ORR [8, 9, 10, 11, 12, 13, 14, 15, 16, 17, 18, 19, 20, 21, 22, 23, 24, 25, 26, 27, 28, 29, 30, 31, 32]. The pooled CRR and ORR for all treatment modalities were 85% (95% CI, 0.76–0.92) and 93% (95% CI, 0.87–0.98) respectively. Four studies used intravitreal methotrexate (IV‐MTX) in combination with systemic methotrexate (sysMTX) for the treatment of VRL with a pooled CRR and ORR of 98% (95% CI, 0.87–1.00). Additionally, four studies employed radiotherapy (RT) combined with methotrexate‐based chemotherapy, resulting in a pooled CRR of 97% (95% CI, 0.89–1.00) and an ORR of 99% (95% CI, 0.94–1.00). Furthermore, three studies employed oral BTKi monotherapy for VRL treatment, resulting in a pooled CRR of 79% (95% CI, 0.49–0.97) and an ORR of 89% (95% CI, 0.76–0.97). Two studies employed MTX‐based chemotherapy, with a pooled CRR of 72% (95% CI, 0.61–0.82) and an ORR of 91% (95% CI, 0.42–1.00). Furthermore, two studies primarily evaluated orbital radiotherapy alone: the study by Isobe et al. included 15 patients who received orbital radiotherapy (excluding 9 patients with WBRT); and the study by Teckie et al. included 18 patients who received orbital radiotherapy (with some receiving subsequent WBRT). Based on these patient data, the pooled CRR was 84% (95% CI, 0.66–0.95) and ORR was 96% (95% CI, 0.71–1.00). Moreover, ten studies utilized various other treatment methods, the detailed results of which are depicted in the forest plot (Figure 1). These findings suggest that conventional treatments like radiotherapy, IV‐MTX, and sysMTX exhibit favorable remission rates for VRL while exploring monotherapy with targeted drugs such as BTKi. The combination of systemic and local therapies seems to lead to higher remission rates.

#### 3.2.2 Survival Data

**FIGURE 2:**
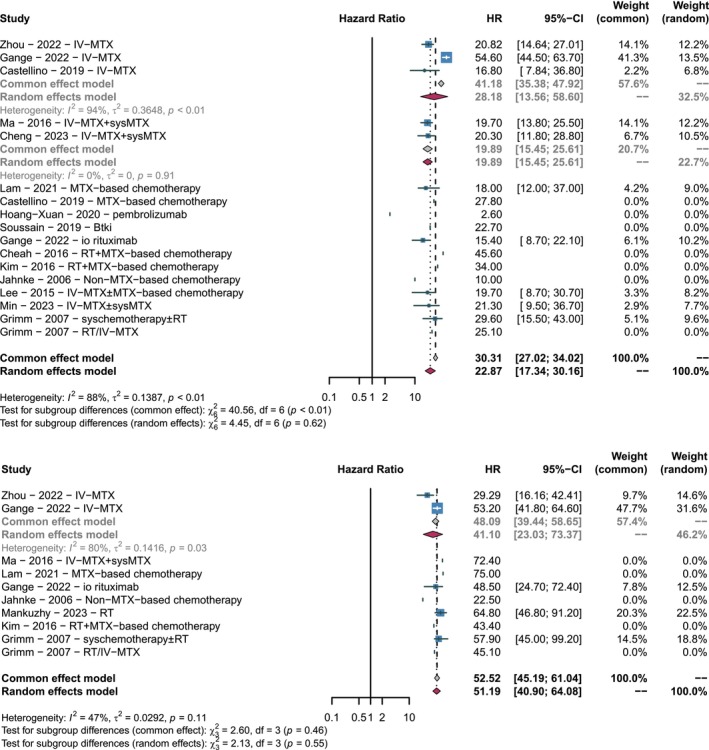
Forest plot illustrating median progression‐free survival (mPFS) and median overall survival (mOS) of various treatment Modalities for vitreoretinal lymphoma (VRL). (Top section) The mPFS of various studies. (Bottom section) The mOS of various studies.

The 18 articles provided specific survival outcomes, including median progression‐free survival (mPFS), median overall survival (mOS), 1‐year progression‐free survival (1‐year PFS), 2‐year progression‐free survival (2‐year PFS), 1‐year overall survival (1‐year OS), and 2‐year overall survival (2‐year OS) [8, 12, 13, 18, 20, 21, 25, 26, 33, 34, 35, 36, 37, 38, 39, 40, 41, 42]. Figure 2, the forest plot, summarizes the mPFS and mOS of all treatment modalities. The pooled total mPFS was 22.87 months (95% CI, 17.34–30.16), with a pooled mPFS of 28.18 months (95% CI, 13.56–58.60) for three studies using IV‐MTX to treat VRL, and 19.89 months (95% CI, 15.45–25.61) for two studies using IV‐MTX + sysMTX. The overall pooled mOS for all treatment modalities was 51.19 months (95% CI, 40.90–64.08), with a pooled mOS of 41.10 months (95% CI, 23.03–73.37) for two studies using IV‐MTX. Additionally, we summarized the 1‐year and 2‐year survival outcomes, with the corresponding forest plots in Figures S2 and S3. The pooled 1‐year PFS was 83% (95% CI, 0.77–0.89), 2‐year PFS was 58% (95% CI, 0.49–0.67), pooled 1‐year OS was 92% (95% CI, 0.87–0.97), and 2‐year OS was 80% (95% CI, 0.74–0.88).

**FIGURE 3:**
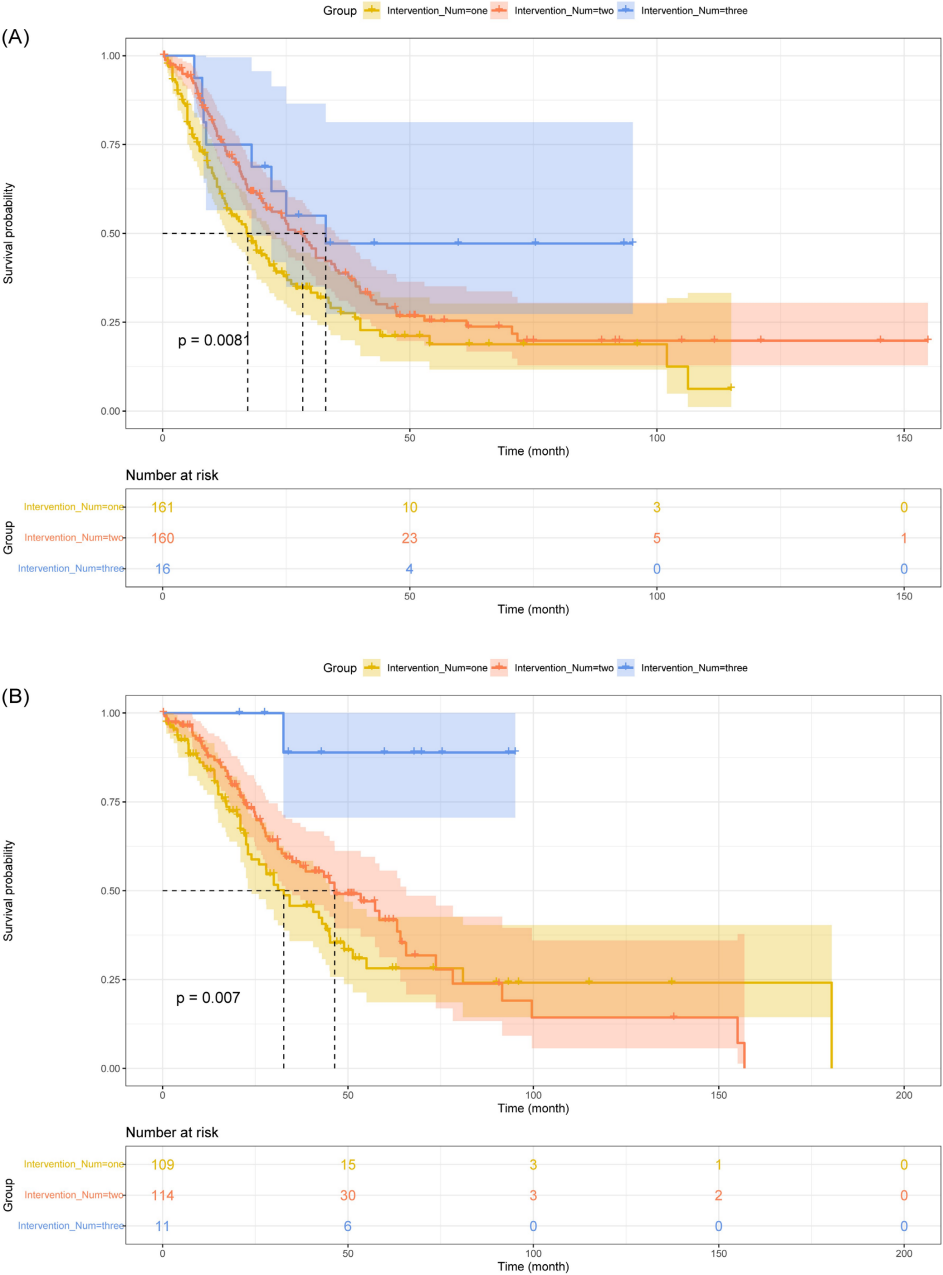
Survival curves comparing survival outcomes based on the number of interventions in treating vitreoretinal lymphoma (VRL). (A) Progression‐free survival (PFS). (B) Overall survival (OS).

**FIGURE 4:**
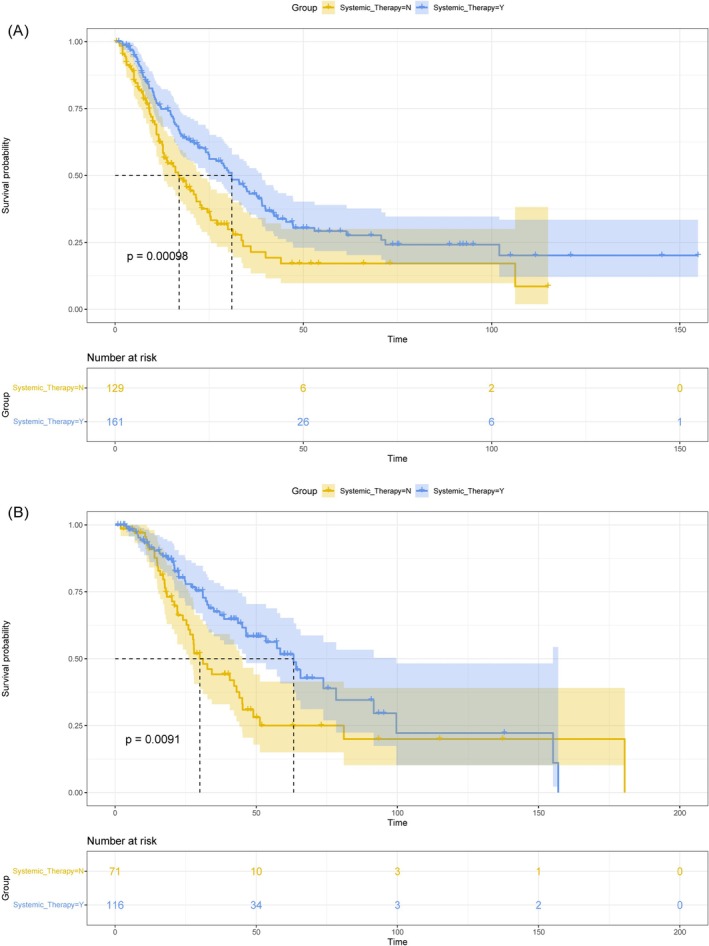
Survival curves comparing survival outcomes with systemic therapy versus no systemic therapy in treating vitreoretinal lymphoma (VRL). (A) Progression‐free survival (PFS). (B) Overall survival (OS).

Furthermore, some articles also provided clinical data [9, 10, 12, 14, 15, 16, 17, 22, 24, 27, 28, 32, 40, 43, 44] or extractable survival curves [21, 25, 37, 38, 39, 41, 42, 45] for patients. After extracting and synthesizing this data, we conducted analyses based on the number of interventions, the use of systemic therapy, age, and whether both eyes were affected. Figure 3 illustrates the significant differences in PFS and OS for varying numbers of interventions in treating VRL (*p*‐values of 0.0081 and 0.007, respectively), demonstrating prolonged PFS and OS with increasing intervention numbers. Figure 4 demonstrates significant extensions in PFS and OS with systemic therapy compared to no systemic therapy (*p* = 0.00098 and *p* = 0.0091, respectively). Moreover, age, sex, and whether both eyes were affected showed no significant differences in post‐treatment survival time (*p* > 0.05). Specific survival curves can be found in Figures [Link], [Link], [Link]. These results indicate that although the overall survival time for VRL is relatively long, the recurrence within a short period cannot be ignored. Therefore, combining multiple regimens and employing systemic therapy may be the primary choice to prolong recurrence‐free and overall survival time.

## 4 Discussion

Currently, there is an absence of standardized treatment protocols for VRL. Common treatment regimens identified in our study encompass IV‐MTX ± systemic HD‐MTX, systemic HD‐MTX alone, radiotherapy ± systemic HD‐MTX, IV‐MTX + systemic HD‐MTX + radiotherapy, multi‐drug chemotherapy ± IV‐MTX/radiotherapy, and BTK inhibitors. This meta‐analysis reveals that irrespective of the treatment strategy employed, a notably high response rate can be attained. The pooled CRR can attain 85%, while the ORR reaches 93.4%. Notably, the pooled CRR for BTK inhibitor monotherapy stands at a relatively low 78.7%, suggesting that solitary targeted drug regimens might lack efficacy against VRL, given its invasive nature. Nevertheless, current research on targeted drugs remains limited, leaving uncertainty regarding the potential enhancement of response rates through combination therapies. Furthermore, systemic HD‐MTX monotherapy yielded a pooled CRR of merely 72.1%, underscoring the significance of localized treatments in VRL management.

Up to 90% of PVRL patients may eventually progress to CNSL [46], significantly impacting the OS of VRL [46, 47, 48]. Several studies have reported that approximately 65%–85% of VRL patients experience deaths related to CNSL [4]. Variations in reported rates of CNS progression among studies may stem from differences in the inclusion criteria for PVRL patients. Riemens et al. gave a strict definition of PVRL, excluding patients with positive cerebrospinal fluid findings as well as patients in whom the diagnosis of PVRL was confirmed after CNSL had developed and reported a lower incidence of CNS progression, with only 36% of PVRL patients experiencing such progression in their study [41]. A systematic review of CNS progression after PVRL revealed an overall incidence rate of 46% during a median follow‐up of 33 (range 1–166) months [49]. This study encompassed all VRL patients. Despite a mOS of 53 months, significantly higher than PCSNL at approximately 26 months [50], the mPFS was only 23 months. Consistent with current research findings, this suggests that regardless of initial CNS involvement in VRL, the rates of intraocular or CNS progression or recurrence remain relatively high. Furthermore, our research indicates that the 2‐year pooled PFS of 57.6% is significantly lower compared to the 83.6% observed at 1 year, suggesting a notable decline. Consequently, addressing strategies for preventing intraocular and CNS progression, particularly long‐term disease progression or recurrence, and facilitating long‐term PFS in VRL patients is a critical concern. However, controversy persists regarding the efficacy of local treatment, chemotherapy, radiotherapy, or targeted therapy in preventing eye or brain lesions.

IV‐MTX can control intraocular lesions, but it is not sufficient to prevent CNS infiltration. In the study we included, Castellino et al. performed IV‐MTX on 17 VRL patients, with a median PFS of only 16.8 months [38]. Local intraocular treatment may improve the quality of life of VRL patients, but it does not increase survival rates [47].

Systemic HD‐MTX‐based chemotherapy is the standard treatment strategy for CNSL. However, HD‐MTX does not appear to have a significant preventive effect on the progression of CNS in VRL and also demonstrates a higher rate of ocular recurrence. In the study included, Lam et al. retrospectively investigated data from VRL patients registered in a national database in France, including 59 PVRL patients who received systemic HD‐MTX treatment [18]. The study found that these patients exhibited a high intraocular recurrence rate, with a median follow‐up period of 61 months, an overall recurrence rate of 71%, an intraocular recurrence rate of 59%, and a CNS recurrence rate of 37% [18]. Similarly, the study by Castellino et al. indicated that 7 PVRL patients receiving HD‐MTX had a median PFS of 27.8 months [38]. Although not conducting a comparative analysis of intraocular or CNS recurrence in these patients, the study confirmed that the combination of IV‐MTX and systemic treatment had a longer recurrence‐free survival for CNS compared to a single treatment strategy, with a median follow‐up of 2.8 years [38]. The IV‐MTX + HD‐MTX group had no CNS recurrence, but there was no difference in OS among the three groups [38]. It is evident that the combination of IV‐MTX and systemic HD‐MTX appears to have certain advantages in prolonging PFS. However, Riemens et al. concluded that different treatment strategies (intraocular therapy alone, systemic therapy, intraocular and systemic combination therapy) do not lead to differences in CNS progression rate, intraocular recurrence, and overall survival [41]. Similarly, a retrospective study of 21 PIOL patients indicated that the combination of IV‐MTX and HD‐MTX did not provide a significant advantage in terms of recurrence rate and recurrence time [13]. Although the authors did not provide an analysis for different conclusions, it is worth noting that in Castellino et al.'s study, a higher proportion of patients received high‐dose chemotherapy or ASCT consolidation therapy. Our findings align with those of Riemens et al., where the pooled median PFS of IV‐MTX and systemic HD‐MTX combination therapy was only 19.891 months, and did not show any advantage over a single strategy of IV‐MTX or systemic HD‐MTX. Moreover, the differing conclusions of related studies also indicate the importance of prospective head‐to‐head studies in addressing these contradictions.

Although the status of systemic HD‐MTX remains controversial, our study, utilizing survival analysis of extracted data, revealed associations between OS and PFS with systemic therapy and combination therapies. This underscores the significance of systemic treatment as a crucial method to prevent disease progression or recurrence. A potential explanation for the controversial preventive effect of combined intraocular and systemic treatments on CNS progression lies in the selection of chemotherapy regimens. This implies that PVRL patients with solely ocular involvement might benefit from selecting PCNSL‐like regimens to lower disease progression or recurrence rates compared to systemic HD‐MTX monotherapy, such as HD‐MTX combined with cytarabine [51], R‐MPV [45], or R‐MBVP [52]. However, it is crucial to remain vigilant as unnecessary intensified treatment may result in chemotherapy‐related adverse events. Furthermore, adverse consequences associated with future CNS lesions might constrain feasible treatment strategies. Hence, recent research by Dalvin et al. underscores the significance of personalized treatment approaches [4]. A retrospective multicenter cohort study, comparing the risk of CNSL between unilateral and bilateral VRL, demonstrated that patients initially presenting with unilateral VRL and those initially treated with systemic chemotherapy for isolated eye diseases had lower likelihoods of developing CNSL [4]. Nonetheless, the initial systemic chemotherapy for isolated eye diseases is not linked to a decrease in all‐cause mortality or CNSL mortality risk [52]. Our study found no associations between age, gender, or bilateral involvement and PFS or OS. Similarly, another meta‐analysis indicated no disparity in CNS progression between bilateral and unilateral cases [49]. While controversy persists regarding CNS progression in unilateral versus bilateral involvement, Dalvin et al.'s study suggests that systemic treatment may confer benefits for VRL patients with monocular involvement [4]. Varied treatment strategies for unilateral and bilateral cases could result in divergent outcomes in CNS progression. Additionally, VRL patients with differing affected areas tend to receive more personalized treatment. Regrettably, there remains no definitive solution regarding CNS prevention for patients with bilateral involvement.

After conducting a systematic evaluation of the included studies, we have identified radiotherapy combined with systemic treatment as having significant potential in the treatment of VRL. Kaburaki et al. conducted a study involving 11 Primary Intraocular Lymphoma (PIOL) patients who received IV‐MTX combined with R‐MPV induction therapy, followed by consolidation with cytarabine and reduced‐dose whole‐brain radiotherapy (rdWBRT), resulting in a 4‐year PFS rate of up to 72.7% [24]. Despite Magnetic Resonance Imaging (MRI) revealing a significant increase in white matter abnormalities during the last 4 years of rdWBRT, only one patient experienced mild cognitive impairment [24]. In a similar study regimen, 11 PIOL patients received R‐MPV treatment, followed by binocular radiotherapy and consolidation high‐dose cytarabine therapy [25]. The median PFS was 45.7 months, with a 2‐year PFS of 63.6% [25].

With the emergence of diverse targeted new drugs, endeavors to address PCNSL using BTK inhibitors, immune modulators, and immune checkpoint inhibitors have all yielded favorable therapeutic outcomes. Nevertheless, research regarding the application of targeted therapeutic drugs in VRL remains scarce, particularly concerning their combination with cytotoxic chemotherapy or other multidrug therapy regimens. Limited understanding persists regarding the management of VRL and the prevention of CNS recurrence. However, due to the success in PCNSL treatment, we believe that targeted therapies are worth exploring more in the field of VRL in the future.

Our research also has some limitations: (1) Lack of randomized controlled studies or head‐to‐head studies; (2) The sample size of published studies is generally small, and the follow‐up time is relatively short; (3) Due to the limited number of studies and variations in clinical treatment protocols, conducting more detailed subgroup analyses is not feasible; (4) Different studies employ varying definitions of VRL and inclusion criteria; (5) Due to language constraints, only English literature was included in this study.

## 5 Conclusions

VRL is a subtype of CNSL, with nearly all patients facing the risk of eventual central progression. Enhancing remission rates, extending survival, and refining prognosis constitute critical concerns for clinicians. Our meta‐analysis of extant research reveals that current treatment strategies yield superior remission rates and prolonged OS in VRL patients. However, PFS remains suboptimal, with early relapse and progression noted in the majority of studies. These challenges necessitate investigation through large‐sample prospective randomized controlled trials. Pursuing the selection of suitable chemotherapy regimens for both local and systemic therapy is crucial to forestall early progression. We anticipate that future availability of more comprehensive data will aid clinicians in enhancing the application of treatment options for VRL.

## Author Contributions

**Jing Gao:** software, writing – original draft, writing – review and editing, visualization, data curation, methodology. **Li‐wei Lv:** writing – original draft, writing – review and editing, methodology, data curation. **Jing Yang:** writing – review and editing. **He‐nan Wang:** writing – review and editing. **Liang Wang:** supervision, writing – review and editing, funding acquisition, conceptualization.

## Conflicts of Interest

The authors declare no conflicts of interest.

## Supporting information


**Figure S1.** Flow diagram of study selection.

**Figure S2.** Forest plot comparing progression‐free survival (PFS) rates at different timepoints for vitreoretinal lymphoma (VRL). Top section: pooled 1‐year PFS rates across included studies. Bottom section: pooled 2‐year OS rates across included studies.

**Figure S3.** Forest plot comparing overall survival (OS) rates at different timepoints for vitreoretinal lymphoma (VRL). Top section: pooled 1‐year OS rates across included studies. Bottom section: pooled 2‐year OS rates across included studies.

**Figure S4.** Survival curves stratified by age group in vitreoretinal lymphoma (VRL) patients. (A) Progression‐free survival (PFS). (B) Overall survival (OS).

**Figure S5.** Survival curves stratified by gender group in vitreoretinal lymphoma (VRL) patients. (A) Progression‐free survival (PFS). (B) Overall survival (OS).

**Figure S6.** Survival curves stratified by whether both eyes are affected in treating vitreoretinal lymphoma (VRL) patients. (A) Progression‐free survival (PFS). (B) Overall survival (OS).

**Table S1.** The search strategy for each database.

## Data Availability

The data generated in this study are available within the article and are available from the corresponding author upon reasonable request.
