# Supplementary material for: Exploration of the Optimal Treatment Modality for Vitreoretinal Lymphoma: A PRISMA Compliant Meta‐Analysis and Systematic Review
Source: Cancer Med. 2025 Jul 30;14(15):e71092. doi: 10.1002/cam4.71092 (PMC12308318; doi:10.1002/cam4.71092)
Supplement: Supplementary file 1 — Figure S1. Flow diagram of study selection. [file CAM4-14-e71092-s006.pdf]

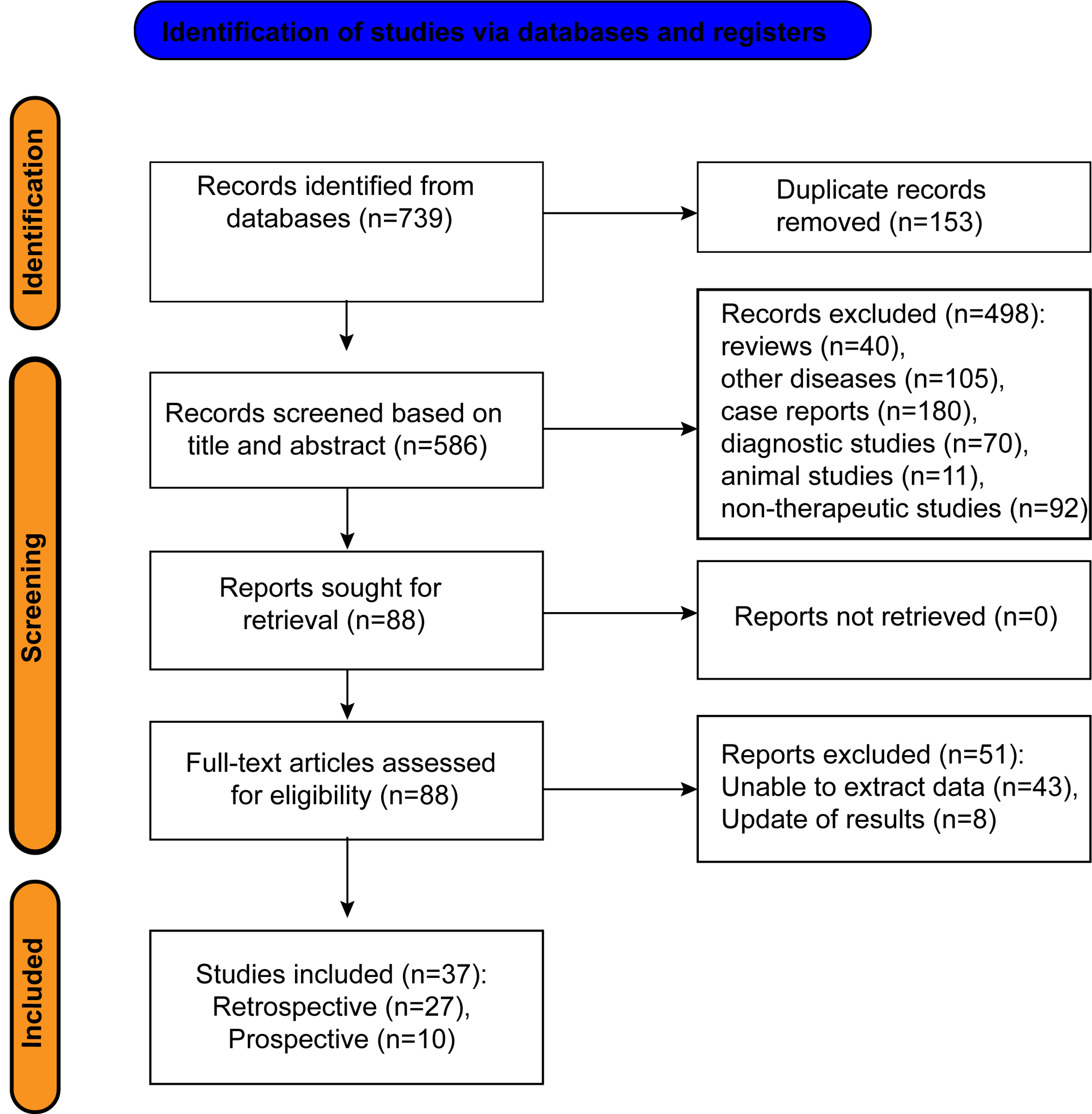
Identification of studies via databases and registers
Identification
Screening
Included
Records identified from databases (n=739)
Duplicate records removed (n=153)
Records screened based on title and abstract (n=586)
Records excluded (n=498):
reviews (n=40),
other diseases (n=105),
case reports (n=180),
diagnostic studies (n=70),
animal studies (n=11),
non-therapeutic studies (n=92)
Reports sought for retrieval (n=88)
Reports not retrieved (n=0)
Full-text articles assessed for eligibility (n=88)
Reports excluded (n=51):
Unable to extract data (n=43),
Update of results (n=8)
Studies included (n=37):
Retrospective (n=27),
Prospective (n=10)
