# Supplementary figures and images for: Exploration of the Optimal Treatment Modality for Vitreoretinal Lymphoma: A PRISMA Compliant Meta‐Analysis and Systematic Review
Source: Cancer Med. 2025 Jul 30;14(15):e71092. doi: 10.1002/cam4.71092 (PMC12308318; doi:10.1002/cam4.71092)

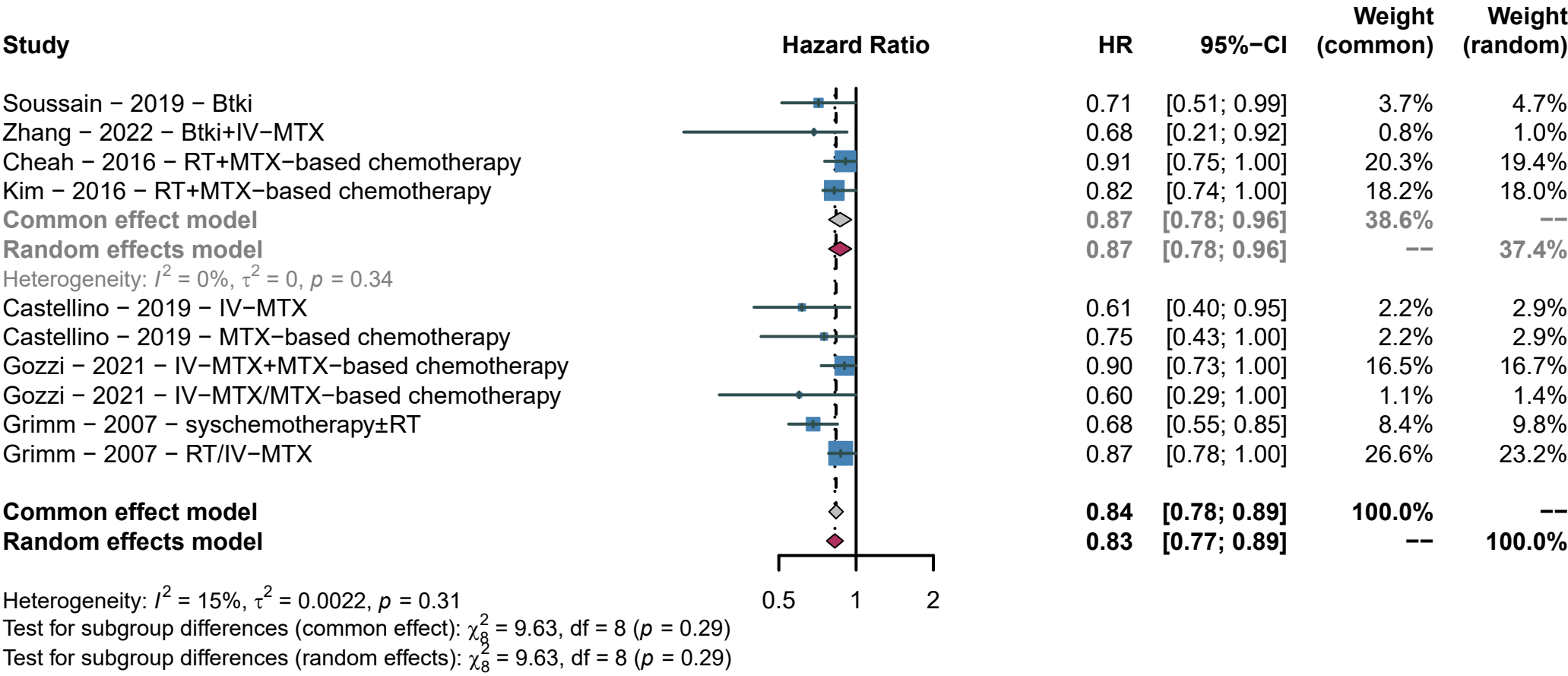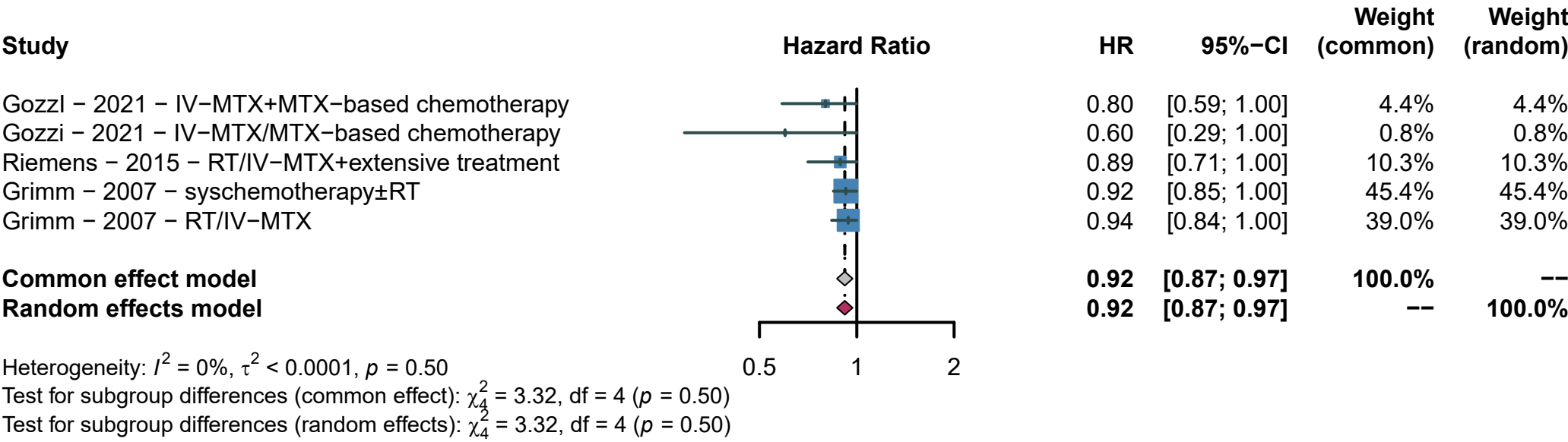

Supplement: Supplementary file 2 — Figure S2. Forest plot comparing progression‐free survival (PFS) rates at different timepoints for vitreoretinal lymphoma (VRL). Top section: pooled 1‐year PFS rates across included studies. Bottom section: pooled 2‐year OS rates across included studies. [file CAM4-14-e71092-s005.pdf]

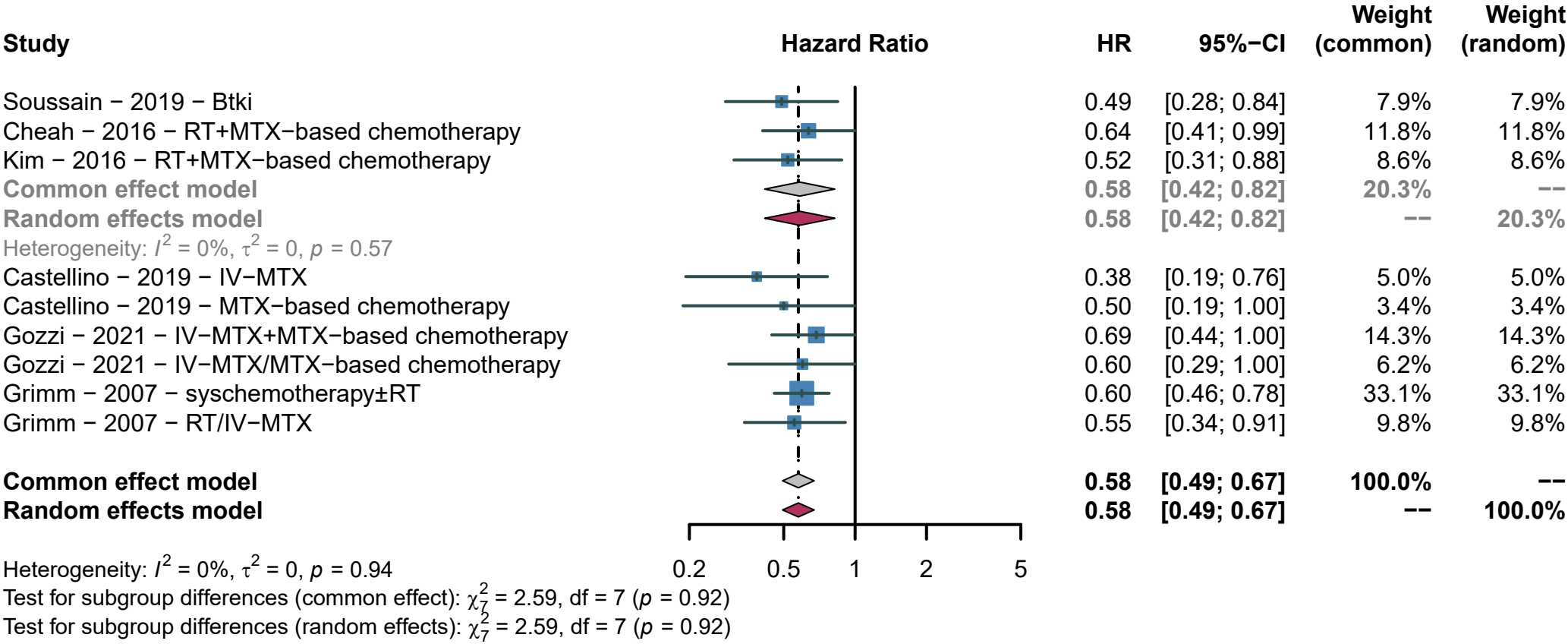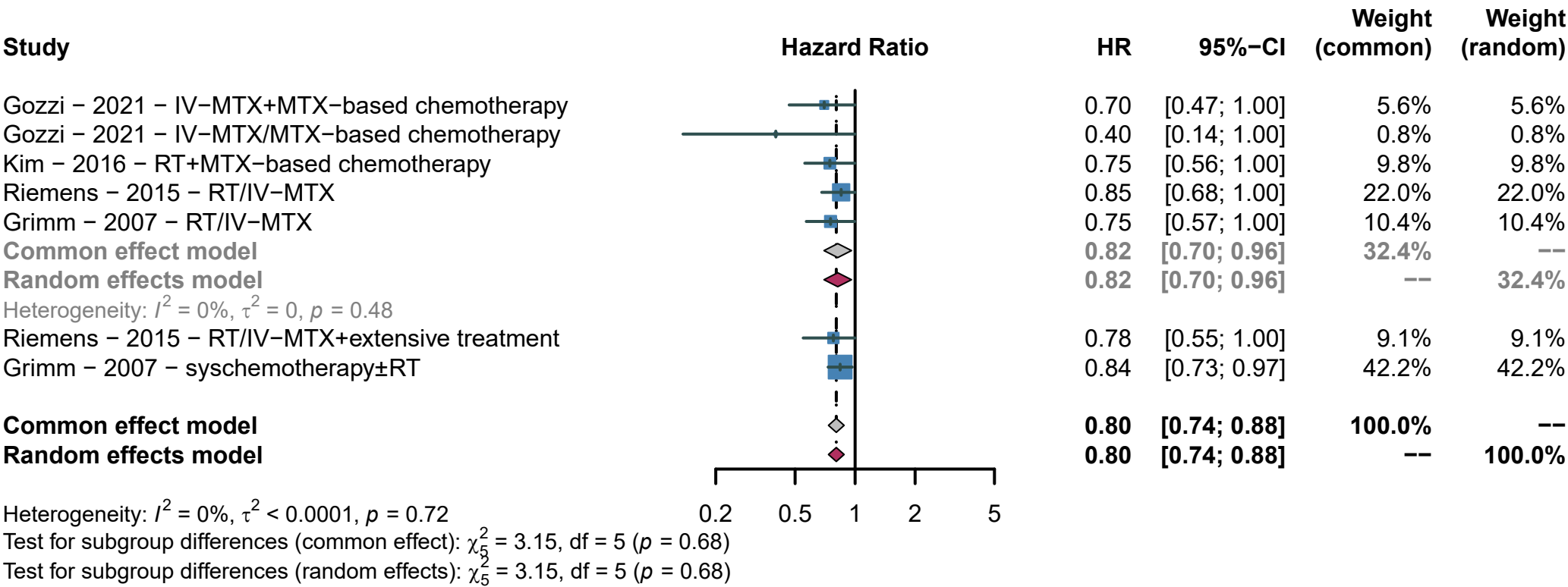

Supplement: Supplementary file 3 — Figure S3. Forest plot comparing overall survival (OS) rates at different timepoints for vitreoretinal lymphoma (VRL). Top section: pooled 1‐year OS rates across included studies. Bottom section: pooled 2‐year OS rates across included studies. [file CAM4-14-e71092-s003.pdf]

(A)

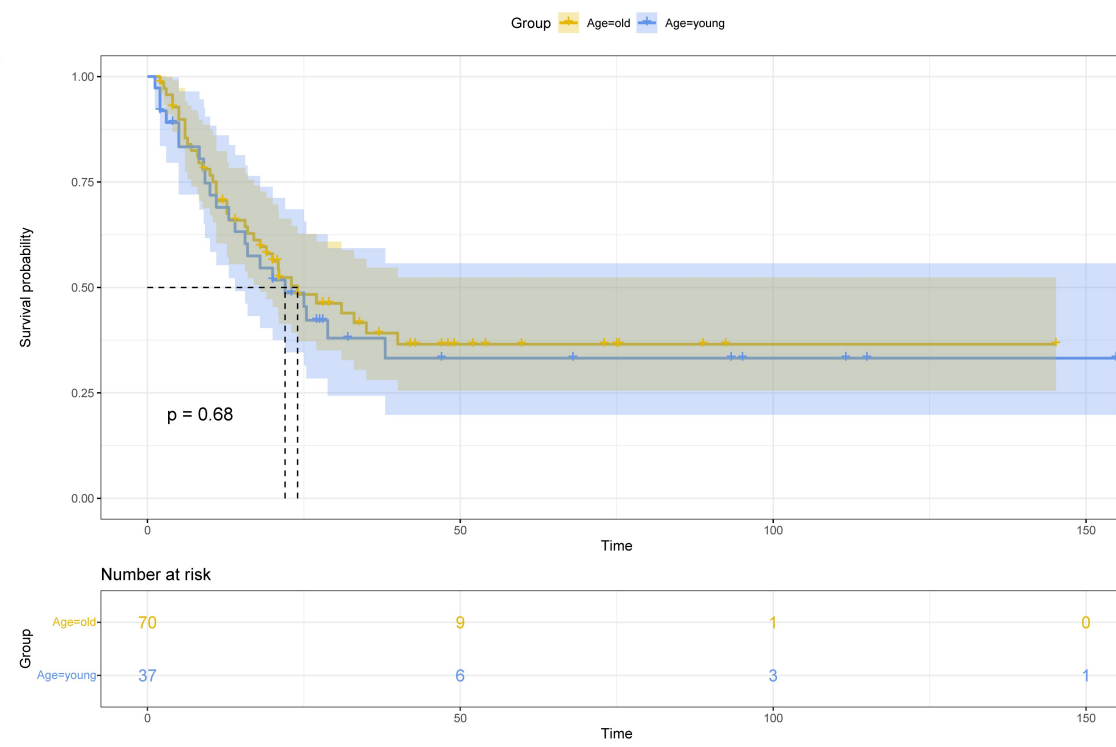

(B)

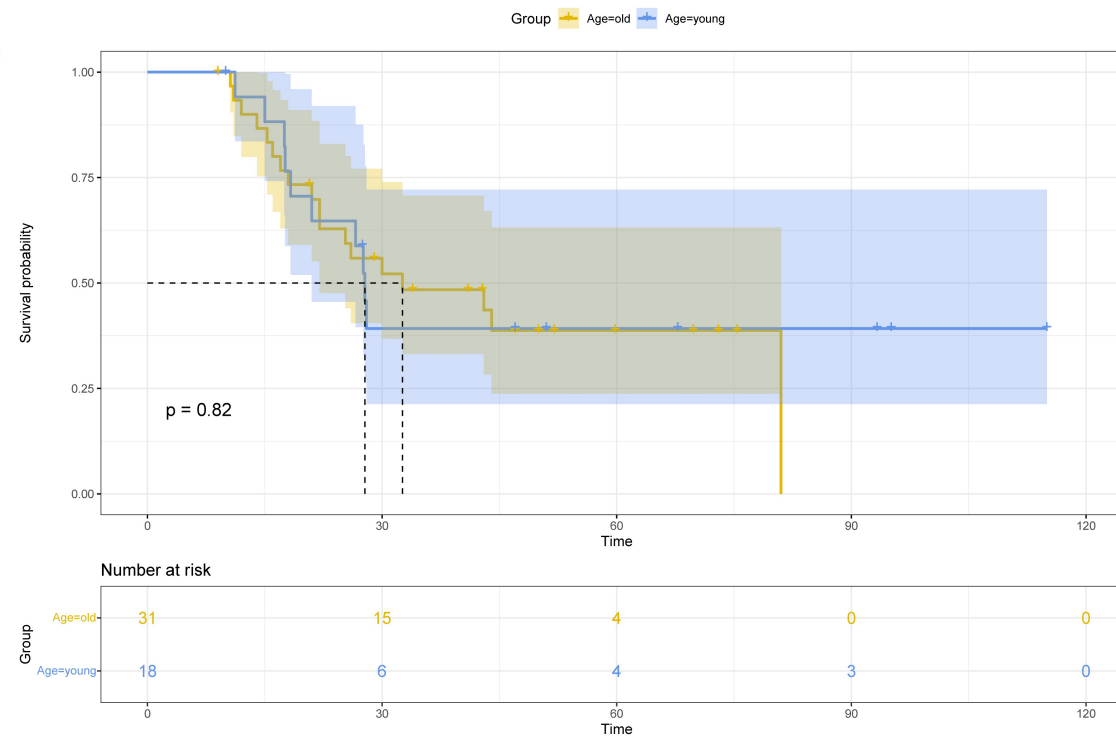

Supplement: Supplementary file 4 — Figure S4. Survival curves stratified by age group in vitreoretinal lymphoma (VRL) patients. (A) Progression‐free survival (PFS). (B) Overall survival (OS). [file CAM4-14-e71092-s002.pdf]

(A)

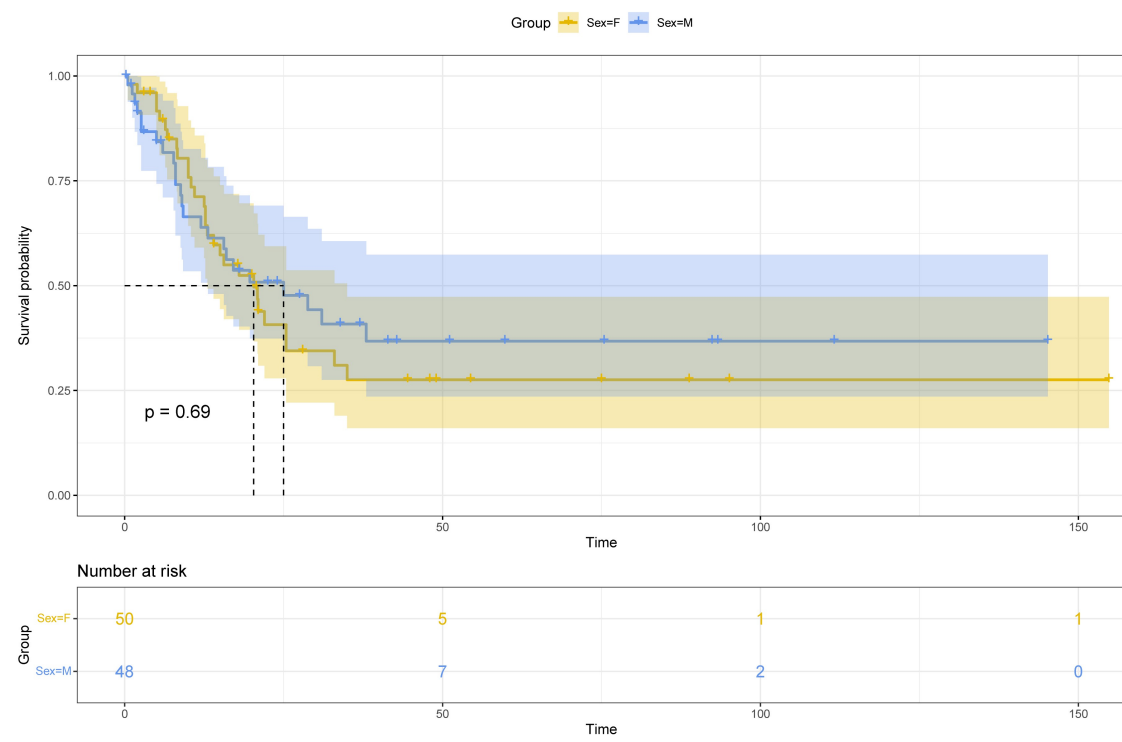

(B)

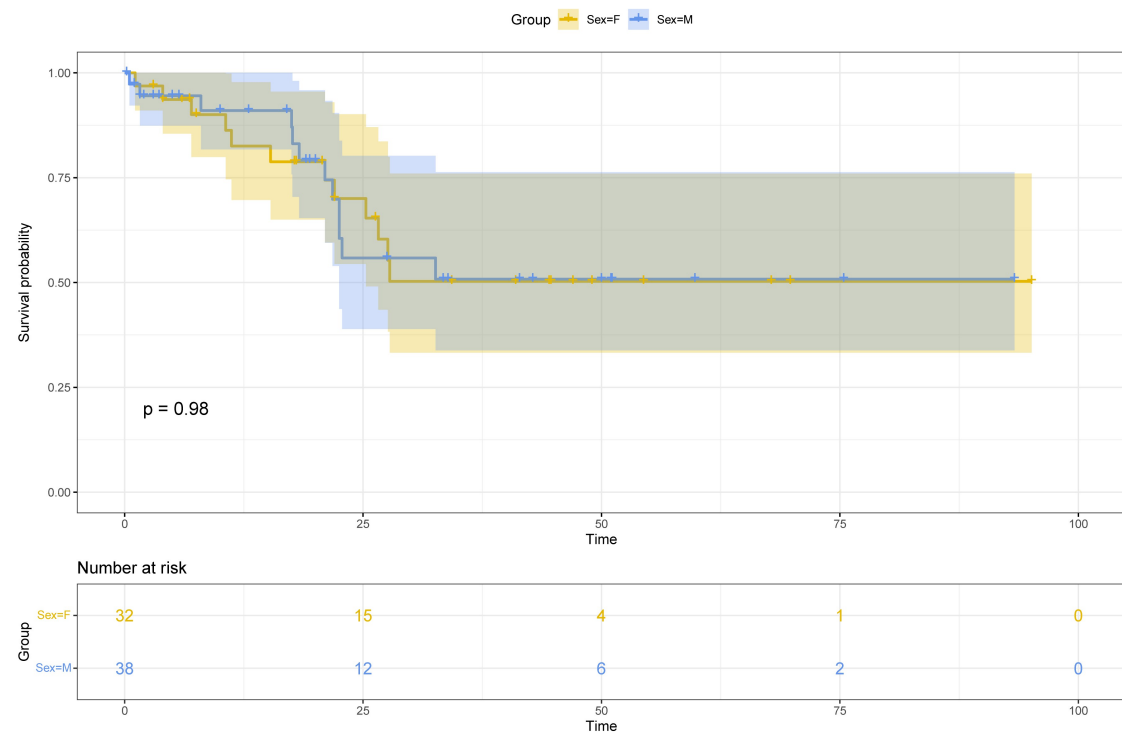

Supplement: Supplementary file 5 — Figure S5. Survival curves stratified by gender group in vitreoretinal lymphoma (VRL) patients. (A) Progression‐free survival (PFS). (B) Overall survival (OS). [file CAM4-14-e71092-s004.pdf]

(A)

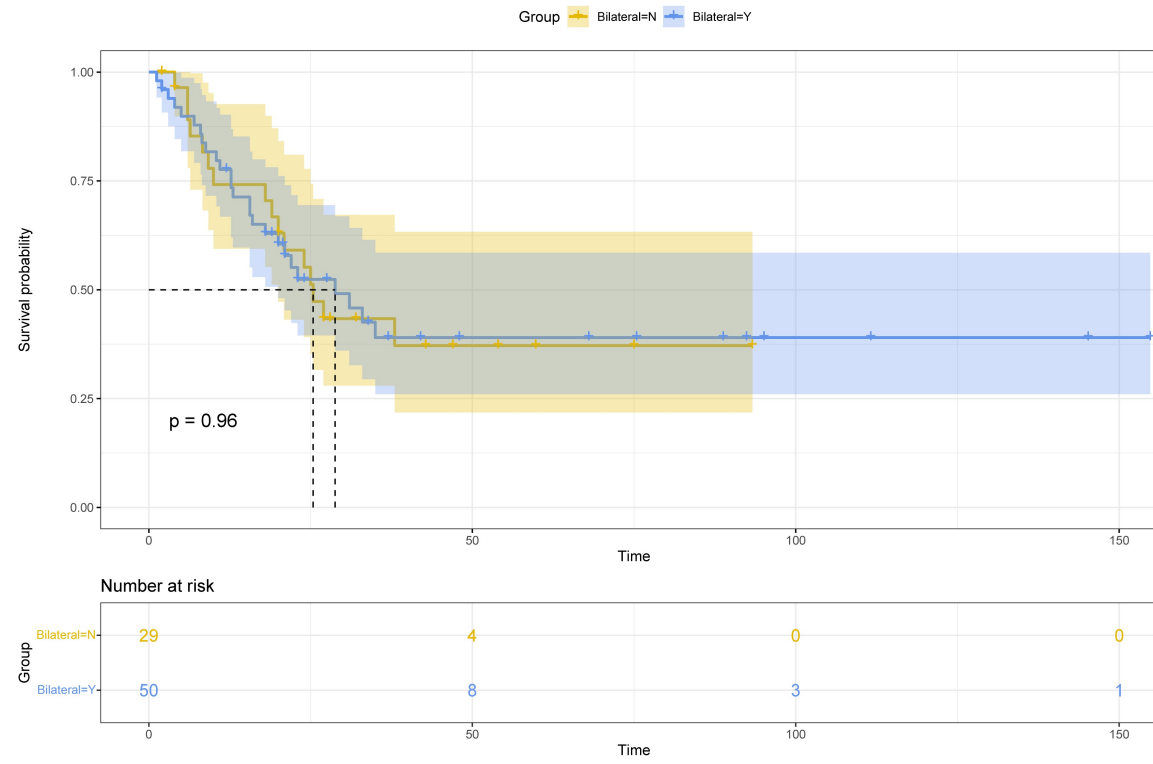

(B)

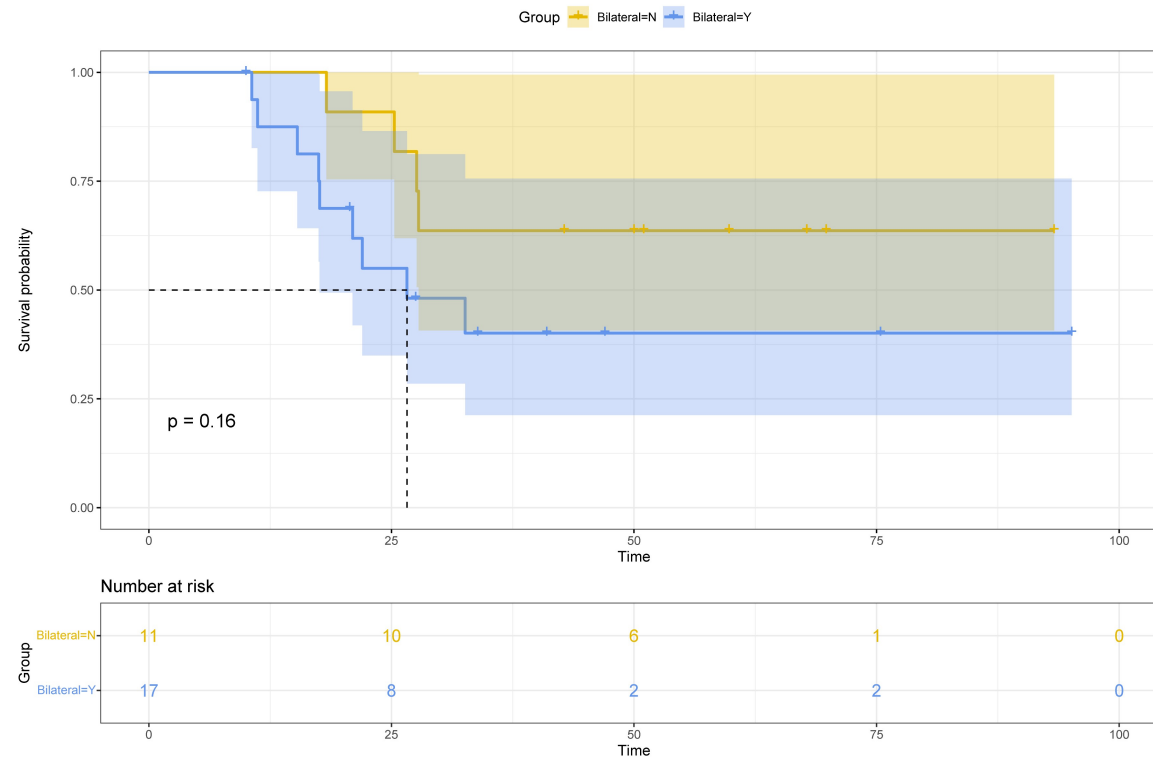

Supplement: Supplementary file 6 — Figure S6. Survival curves stratified by whether both eyes are affected in treating vitreoretinal lymphoma (VRL) patients. (A) Progression‐free survival (PFS). (B) Overall survival (OS). [file CAM4-14-e71092-s001.pdf]
