# Supplementary material for: Exploration of the Optimal Treatment Modality for Vitreoretinal Lymphoma: A PRISMA Compliant Meta‐Analysis and Systematic Review
Source: Cancer Med. 2025 Jul 30;14(15):e71092. doi: 10.1002/cam4.71092 (PMC12308318; doi:10.1002/cam4.71092)
Supplement: Supplementary file 7 — Table S1. The search strategy for each database. [file CAM4-14-e71092-s007.docx]

**Supplementary Table 1**

Pubmed——101：

("PVRL"[Title/Abstract] OR "primary vitreoretinal lymphoma"[Title/Abstract] OR "VRL"[Title/Abstract] OR "Vitreoretinal lymphomas"[Title/Abstract] OR "Primary intraocular lymphoma"[Title/Abstract] OR "Intraocular Lymphomas"[Title/Abstract] OR "lymphoma intraocular"[Title/Abstract] OR "lymphomas intraocular"[Title/Abstract] OR "intraocular lymphoma"[MeSH Terms]) AND ("Eye"[MeSH Terms] OR "Eyes"[Title/Abstract] OR "intraocular"[Title/Abstract] OR "Vitreoretinal"[Title/Abstract] OR "ocular"[Title/Abstract] OR "vitreous retinal"[Title/Abstract]) AND (("clinical"[Title/Abstract] AND "trial"[Title/Abstract]) OR "clinical trials as topic"[MeSH Terms] OR "clinical trial"[Publication Type] OR "random*"[Title/Abstract] OR "random allocation"[MeSH Terms] OR "therapeutic use"[MeSH Subheading]) NOT ("Review"[Publication Type] OR "systematic review"[Publication Type] OR "case report"[Title/Abstract])

Embase——440：

('pvrl':ti,ab,kw OR 'primary vitreoretinal lymphoma':ti,ab,kw OR 'vrl':ti,ab,kw OR 'vitreoretinal lymphomas':ti,ab,kw OR 'primary intraocular lymphoma':ti,ab,kw OR 'intraocular lymphomas':ti,ab,kw OR 'lymphoma, intraocular':ti,ab,kw OR 'lymphomas, intraocular':ti,ab,kw OR 'intraocular lymphoma'/exp OR 'primary vitreoretinal lymphoma'/exp) AND ('eye':ti,ab,kw OR 'eyes':ti,ab,kw OR 'intraocular':ti,ab,kw OR 'vitreoretinal':ti,ab,kw OR 'ocular':ti,ab,kw OR 'vitreous retinal':ti,ab,kw) AND ('clinical trial'/exp OR 'therapy'/exp OR 'Therapeutic':ti,ab,kw OR 'Therapeutics':ti,ab,kw OR 'Therapy':ti,ab,kw OR 'Therapies':ti,ab,kw OR 'Treatment':ti,ab,kw OR 'Treatments':ti,ab,kw) NOT ('Review'/it OR ‘case report’:ti,ab,kw) NOT (('animal experiment'/exp OR 'animal model'/exp OR 'nonhuman'/exp) NOT 'human'/exp)

Scopus_139:

( TITLE-ABS-KEY ( "PVRL" OR "primary vitreoretinal lymphoma" OR "VRL" OR "Vitreoretinal lymphomas" OR "primary intraocular lymphoma" OR "intraocular lymphomas" OR "lymphoma, intraocular" OR "lymphomas, intraocular") AND TITLE-ABS-KEY ( "Eye" OR "Eyes" OR "intraocular" OR "Vitreoretinal" OR "ocular" OR "vitreous retinal" )) AND TITLE-ABS-KEY (“clinical trial” OR “Intervention Study” OR “Therapeutics” OR “Therapeutic” OR “Therapy” OR “Therapies” OR “Treatment” OR “Treatments”) NOT TITLE-ABS-KEY("Review" OR "Case Report" OR "case of")
